# Effective Stimuli for Constructing Reliable Neuron Models

**DOI:** 10.1371/journal.pcbi.1002133

**Published:** 2011-08-18

**Authors:** Shaul Druckmann, Thomas K. Berger, Felix Schürmann, Sean Hill, Henry Markram, Idan Segev

**Affiliations:** 1 Interdisciplinary Center for Neural Computation, Hebrew University of Jerusalem, Jerusalem, Israel; 2 Edmond and Lily Safra Center for Brain Sciences and Department of Neurobiology, Institute of Life Sciences, Hebrew University of Jerusalem, Jerusalem, Israel; 3 Brain Mind Institute, Ecole Polytechnique Fédérale de Lausanne (EPFL), Lausanne, Switzerland; Université Paris Descartes, Centre National de la Recherche Scientifique, France

## Abstract

The rich dynamical nature of neurons poses major conceptual and technical challenges for unraveling their nonlinear membrane properties. Traditionally, various current waveforms have been injected at the soma to probe neuron dynamics, but the rationale for selecting specific stimuli has never been rigorously justified. The present experimental and theoretical study proposes a novel framework, inspired by learning theory, for objectively selecting the stimuli that best unravel the neuron's dynamics. The efficacy of stimuli is assessed in terms of their ability to constrain the parameter space of biophysically detailed conductance-based models that faithfully replicate the neuron's dynamics as attested by their ability to generalize well to the neuron's response to novel experimental stimuli. We used this framework to evaluate a variety of stimuli in different types of cortical neurons, ages and animals. Despite their simplicity, a set of stimuli consisting of step and ramp current pulses outperforms synaptic-like noisy stimuli in revealing the dynamics of these neurons. The general framework that we propose paves a new way for defining, evaluating and standardizing effective electrical probing of neurons and will thus lay the foundation for a much deeper understanding of the electrical nature of these highly sophisticated and non-linear devices and of the neuronal networks that they compose.

## Author Summary

Neurons perform complicated non-linear transformations on their input before producing their output - a train of action potentials. This input-output transformation is shaped by the specific composition of ion channels, out of the many possible types, that are embedded in the neuron's membrane. Experimentally, characterizing this transformation relies on injecting different stimuli to the neuron while recording its output; but which of the many possible stimuli should one apply? This combined experimental and theoretical study provides a general theoretical framework for answering this question, examining how different stimuli constrain the space of faithful conductance-based models of the studied neuron. We show that combinations of intracellular step and ramp currents enable the construction of models that both replicate the cell's response and generalize very well to novel stimuli e.g., to “noisy” stimuli mimicking synaptic activity. We experimentally verified our theoretical predictions on several cortical neuron types. This work presents a novel method for reliably linking the microscopic membrane ion channels to the macroscopic electrical behavior of neurons. It provides a much-needed rationale for selecting a particular stimulus set for studying the input-output properties of neurons and paves the way for standardization of experimental protocols along with construction of reliable neuron models.

## Introduction

Ever since the seminal study of Hodgkin and Huxley [1] on the biophysical basis of the squid giant axon action potential, conductance-based models (CBMs) have provided a critical connection between the microscopic level of membrane ion channels and the macroscopic level of signal flow in neuronal circuits. Indeed, as we have sought to further our understanding of single neuron and network computation [2], [3], CBMs have become one of the powerful computational approaches in Neuroscience [4], [5], [6], [7]. They have been of great assistance in incorporating diverse experimental data under a coherent, quantitative framework and for interpreting experimental results in a functionally meaningful way [8], [9], [10], [11], [12], [13], [14], [15], [16], [17], [18], [19]. Considering the dramatic advancements in our knowledge of single neurons and neural circuits along with the equally impressive increase in computing power during the last decade, CBMs can be expected to become of even greater utility than they already are today [20], [21], [22].

The most fundamental difficulty in accurately modeling neurons stems from the fact that their electrical behavior arises from the complex interaction of a large number of non-linear elements – the membrane ion channels [10], [23]. Furthermore, the identity and density of different ion channels vary from neuron to neuron and cannot presently be directly determined experimentally. Instead, these are treated as free parameters that are typically constrained by an iterative process of comparison between a set of experimental recordings (e.g. voltage response to current-clamp steps) and the model's responses until a close resemblance is found. Yet successful matching of model response to a given target experimental data set is not, in and of itself, sufficient to establish the validity of a model, as complex models with numerous parameters run the risk of systematic biases (or errors) in the estimation of their parameters, i.e. over-fitting. Specifically, such a bias may not be apparent in the accuracy of matching the response to stimuli used to construct the model, but may be revealed by further testing of the model's generalization to different conditions. One can imagine many different such tests: predicting the response to pharmacological manipulation, examining the stability of the model to small perturbations of model parameter values, etc.

Here we describe the application of a particularly intuitive yet powerful measure of generalization: the model's ability to generate an accurate response to a set of current stimuli to which it has not been previously exposed during the model's construction. We favor this form of testing generalization since it gets to the heart of the purpose of conductance-based models (CBMs) – to examine whether the model can indeed be considered a valid approximation of the neuron's underlying dynamics. If that were indeed the case, one would expect the response of the model to match the experimental response not only to the stimuli used to constrain it, but also to different, novel inputs. Moreover, measuring the response to different stimuli is experimentally straightforward. Such measurement of generalization has only been sporadically applied in CBM research papers [24] most likely due to the fact that the vast majority of CBMs studies involved hand tuning of parameters [25], [26] in which clean separation between test sets and generalization sets is difficult due to human involvement. Thus, despite its importance, the quantification of generalization has been lacking from conductance-based neuron modeling.

Since there are many different choices for stimuli that can be used to train and test a model, it is crucial to have a clear way of selecting an optimal (and minimal) set of stimuli (and corresponding targets to be preserved) that will ensure accurate generalization to a wide range of inputs. The present work is the first to have addressed these fundamental principles in order to assess the validity of CBMs in a thorough manner. We experimentally recorded the responses of a variety of cortical neurons to a wide set of different current stimuli (step, ramp and noise currents) each with multiple intensities and many repetitions. We then selected a subset of the experimental data (a training set) to be employed in generating models of the respective cells, using automated multiple objective optimization algorithms, and reserved another set of stimuli to test the accuracy of these models in generalizing to novel stimuli.

We show that, for all neurons tested, CBMs were able to accurately predict stimuli not encountered during the parameter constraining process. Furthermore, by systematically changing the number and type of stimuli used to constrain the models, we determined how each stimulus contributes to the models' predictive power. Notably, models trained solely on responses to step currents were able to accurately predict both the responses to simple stimuli such as ramp current as well as to the responses to physiologically inspired noisy current injections. In contrast, models trained on either ramp or noisy inputs were not as successful in predicting the response to other types of stimuli, i.e. do not generalize well. We discuss the reasons why some stimuli are more successful for estimating the properties of the underlying ion channels than others as well as the implication of this work on the way we understand the process of constraining biophysical neuron models and on the data collection approach required to allow the generation of accurate, predictive CBMs. We believe that our method will become a standard tool for generating *in-silico* models for a variety of neuron types and that these models could then be used in realistic models of large scale neuronal networks.

## Results

**Figure 1:**
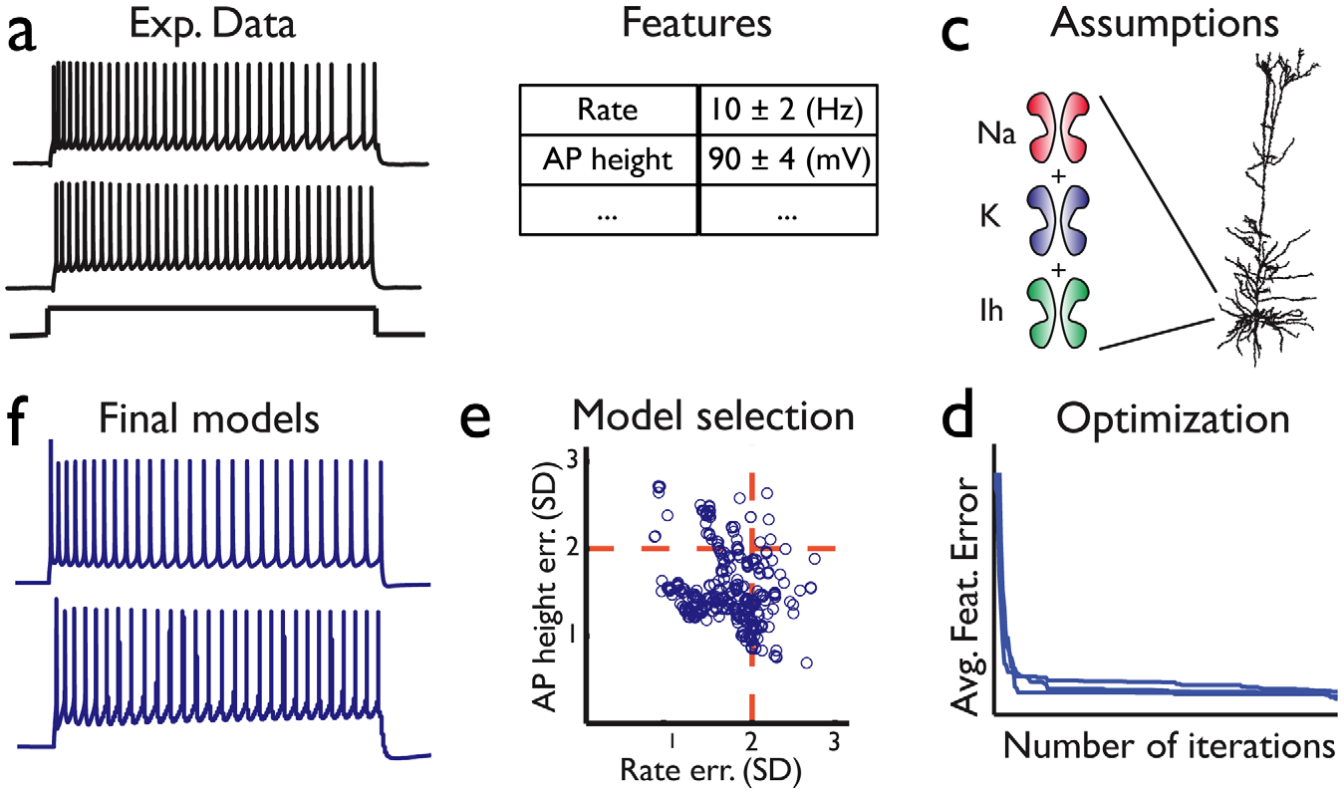
From experimental data to acceptable conductance-based neuron model. (**a**) Data is collected from voltage responses to a set of repeated intracellular current injections (steps, ramps, noise currents) recorded from single cells' somata. Two repetitions of a step current injection are shown. Two traces with fairly large differences were chosen to highlight the variability. (**b**) The voltage traces are characterized using a set of features (e.g. firing rate, height of action potentials). For each feature both the experimental mean and standard deviation (SD) are obtained from 15 repetitions of the same stimulus. (**c**) The generic form of a model to be constrained consists of a reconstructed morphology and an assumed set of membrane ion channels (including their kinetics but not their densities, g_i_). (**d**) A multiple objective, genetic algorithm-based process of stochastic optimization is applied in order to obtain values for g_i_ that minimize the distance between the experimentally measured set of features and those of the model. The convergence of the average error is shown by the blue curves, one curve for each of three independent applications of the model constraining procedure (**e**) For the many possible solutions at the final iteration, a selection criterion of two experimental SDs in each feature is used for choosing a subset of solutions (sets of g_i_ values); these are considered acceptable models. Shown are two out of the six features considered for step stimuli (see Materials and methods) (f) An example of the response of two different successful models to a step current input as in a. Two models with fairly large differences were chosen to highlight the variability. The reconstructed L5 pyramidal cell shown in c is used throughout Figures 1–6.

The process of constraining (training) the CBMs is portrayed in Figure 1 and explained in detail in the **Materials and Methods** section. Briefly, from the experimental data, voltage responses to suprathreshold current inputs, (Figure 1a) we first extract a set of features (Figure 1b). We then obtain the reconstructed morphology of the neuron and assume a set of membrane conductances (ion currents) to be present in the neuron's soma (Figure 1c). In the present study we assumed that the modeled cortical cells contain: Transient sodium channel-Nat, Delayed potassium channel-Kd, Slow inactivating persistent potassium channel-Kp, fast non-inactivating potassium channel Kv3.1 channel, high-voltage-activated calcium channel Ca, calcium dependent K channel - SK, Hyperpolarization-activated cation current – Ih, M-type potassium channel Im (for full details see **Materials and Methods**). In the interests of simplicity, and since recordings were performed in the soma, we assumed the neuron's dendrites to be passive. We then run an optimization algorithm (a Multiple Objective Optimization (MOO) algorithm [27]) to constrain the values of the maximal conductances of these ion channels and of the passive properties of the neuron. The optimization generates a set of multiple models from which we select for further analysis only the models that pass a selection criterion (Figure 1e). These constitute the final set of acceptable models (Figure 1f).

**Figure 2 pcbi-1002133-g002:**
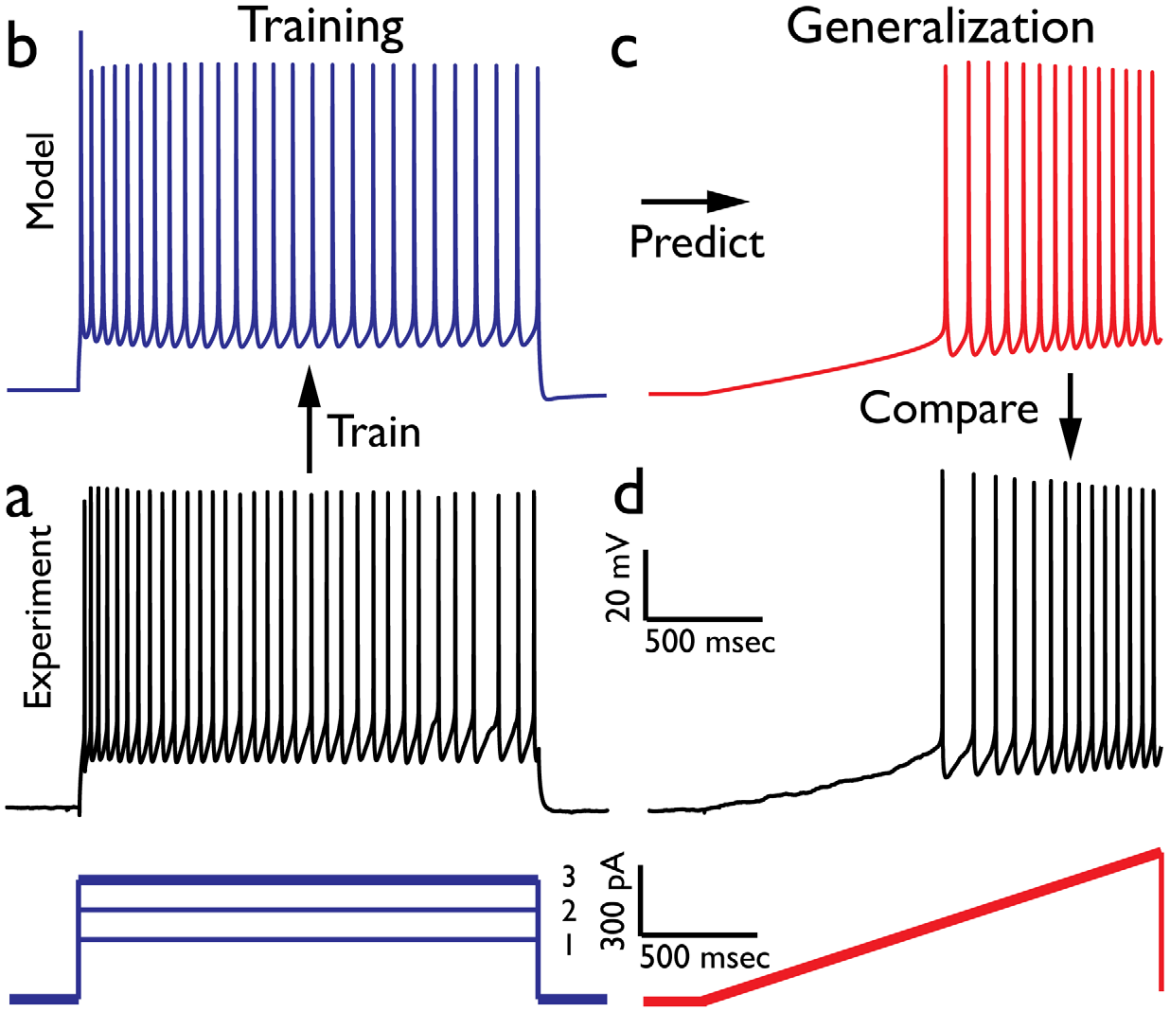
Training and Generalization paradigm. Example - training on responses to step currents and generalizing to responses for ramp currents. (**a**) Experimental voltage responses recorded from rat layer 5 pyramidal cell (depicted in Figure S1c) to three depolarizing current steps (lower blue) are used as the training set; the experimental response to the largest step current, #3, is displayed in black. (**b**) Model response to step current #3 following training on the three current steps. (**c**) Model response (red trace) to a new stimulus, in this case a ramp current (lower red trace in **d**). (**d**) Experimental response to the same ramp current. Comparison between model prediction and experimental data, using feature-based distance functions, enables one to quantify the accuracy of the generalization procedure (see Figure 3). In this case the average feature error was approximately 1.5 in units of the experimental standard deviation.

The procedure of assessing the models' generalization power is depicted in Figure 2. In Figure 2a three suprathreshold step currents (together with the corresponding experimental voltage responses) were injected to a rat layer V pyramidal cell and used as the training set. Model parameters, maximal conductance values for the eight excitable ion channels modeled and the neuron's passive properties, were automatically constrained until the response of the resulting set of models closely matched the experimental data (Figure 2b).

Then, while keeping the model parameters fixed, we applied to the models a new set of stimuli (ramp currents in this example) that were not encountered during the parameter constraining procedure, and recorded the models' voltage response to these new stimuli (Figure 2c, red trace, generalization). Finally, we quantified the degree of resemblance of the model response to that of the corresponding experimental response (Figure 2d). This is quantitatively expressed as the model mismatch, or error, as measured by the feature-based distance between model and experiment in units of experimental standard deviation (SD) [27].

**Figure 3 pcbi-1002133-g003:**
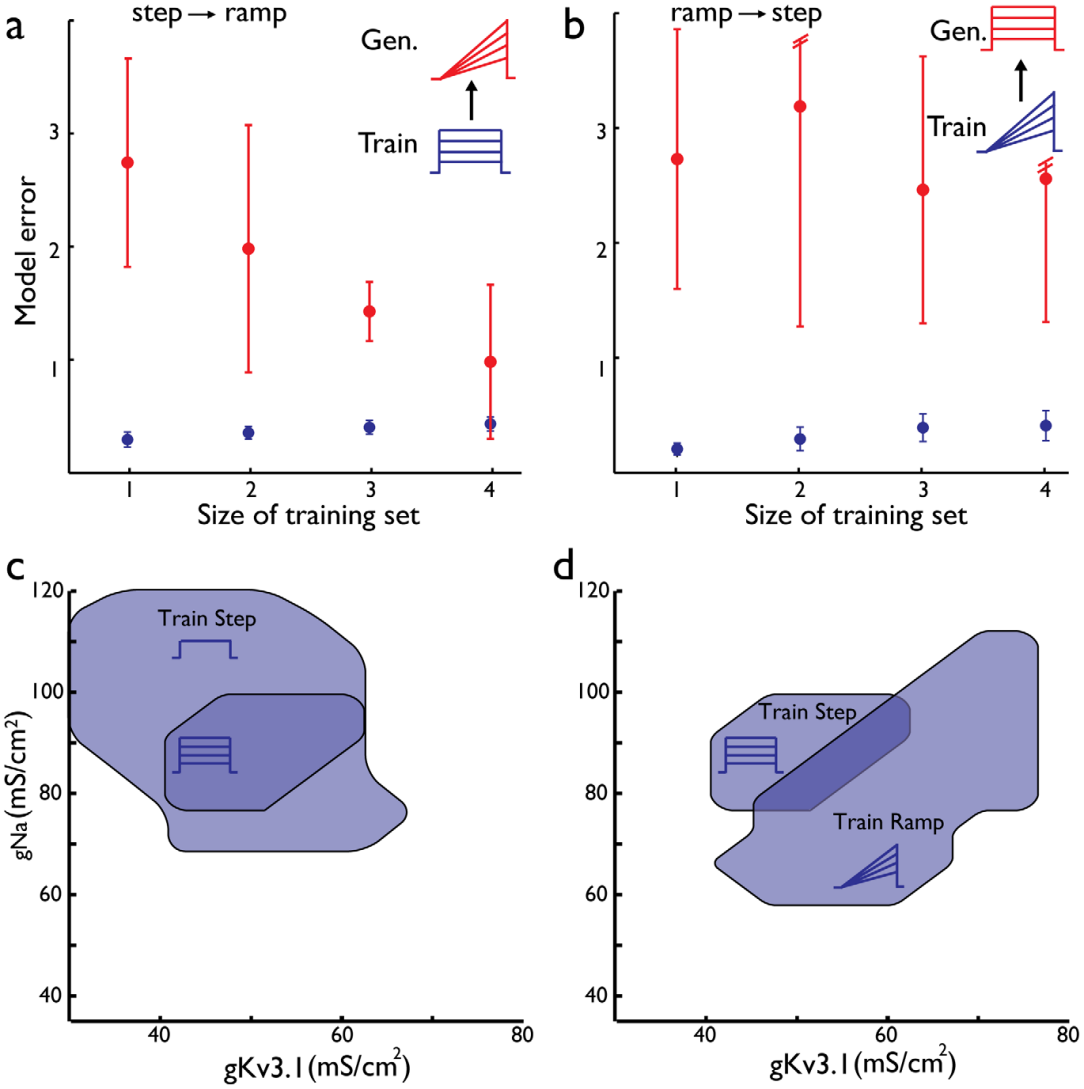
Asymmetric generalization for step and ramp current stimuli. (**a**) Models were trained on step currents and generalization tested on ramp currents. Mean and standard deviation of training error (blue) and generalization (red) for increasing number of stimuli included in the training set. (**b**) Models were trained on ramp stimuli and tested for generalization on step stimuli. (**c**) Space of acceptable solutions for two out of eight ion channel conductances used in this study. Transient sodium (gNa) and fast potassium (gKv3.1) conductances are shown for models trained on one current step (region in light blue) and models trained on four currents steps (dark blue). (**d**) Space of acceptable solutions for both step and ramp stimuli (four stimuli in each case) for the two ion channels depicted in (**c**). The intersection area (darker blue) represents solutions that are consistent with both stimuli types.

Figure 3a depicts the ability of models constrained by responses to step currents to predict (generalize to) the response to suprathreshold ramp current injections. We find that as the size (the number of different step currents) of the training set increases, the average training error between the model and the experimental responses slightly increases (Figure 3a, blue circles). This is expected from learning theory as a model of a given complexity is challenged to fit a growing number of targets [28]. However, the more interesting measure of model accuracy is the error in matching responses to stimuli *not* encountered during the parameter constraining procedure, the generalization error. This error steeply decreases with the size of the training set (Figure 3a, red circles, difference between one and four stimuli, P<0.0001) indicating more accurate, reliable (better constrained) models.

We next turn to constraining models by ramp current injection (Figure 3b). Surprisingly, when attempting to generalize to step currents using models that were trained on ramp currents, an increase on the size of the training set did not yield better generalization for the response to step currents and the distribution of the generalization errors was very broad (Figure 3b).

In order to determine the impact of the nature and number of stimuli used to constrain models on the conductance values of successful solutions, we portray in Figure 3c the spread of parameter values found at the end of the parameter optimization process, as well as simulations of all points on a grid [29]. The spread of solutions consistent with one step stimulus was considerably larger than that of solutions consistent with four (ratio of areas 0.24; Figure 3c, light and dark blue areas for one and four stimuli, respectively, shown in two dimensional space). Note that though visualization is difficult beyond three dimensions, the calculation of the consistency of points on a grid with each stimulus can be readily performed on the high dimensional grid. This reduction in area with increasing number of training set stimuli can be seen for most individual conductance dimensions when considered separately as well (Figure S1a).

When considering models constrained on ramp currents we again find that for most conductances an increase in the number of stimuli leads to reduction in the spread of successful solutions (Figure S1b). However, the relative size of the area of solutions consistent with four ramp and step current stimuli (Figure 3d blue areas marked with stimulus icon) in relation to the area of intersection (Figure 3d dark blue) is much larger for ramp currents than step currents. Thus, a solution chosen at random from those consistent with step currents is far more likely to be in the area of intersection, i.e. to be consistent with responses to both ramp and step currents. This is directly in line with the more successful generalization from step current responses to responses to ramp currents than *vice versa*. We note that the different ways in which stimuli “carve out” zones in parameter space is highly relevant to the problem of solution non-uniqueness and return to this subject in the **Discussion**.

**Figure 4 pcbi-1002133-g004:**
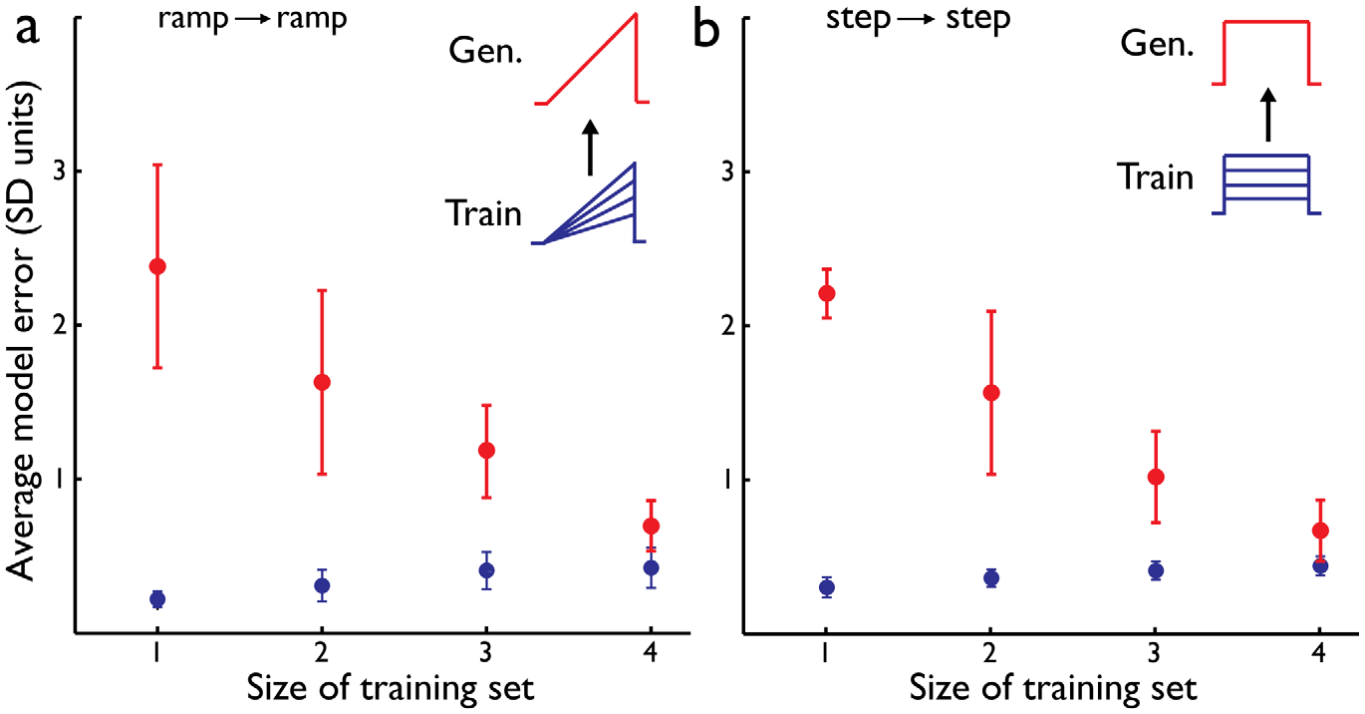
Within stimulus generalization. (**a**) Models were trained on ramp stimuli and tested for generalization on ramp stimuli Mean and standard deviation of training error (blue) and generalization (red) for increasing number of stimuli included in the training set. (**b**) Models were trained on step currents and generalization tested on different intensities of step currents.

To ensure that the asymmetric generalization is not due to an inherent difficulty with constraining models to match responses to ramp stimuli we quantified the ability of models trained on ramp currents to generalize within stimulus, e.g., to other ramp current stimuli not encountered during the parameter constraining procedure. We find that, in contrast to the between stimulus generalization, addition of stimuli results in decreased generalization error (Figure 4a, difference between one and four ramp stimuli, P<0.0001). We compared this to the within stimulus generalization in step currents and found it to be qualitatively similar (Figure 4b, difference between one and four step stimuli, P<0.0001).

**Figure 5 pcbi-1002133-g005:**
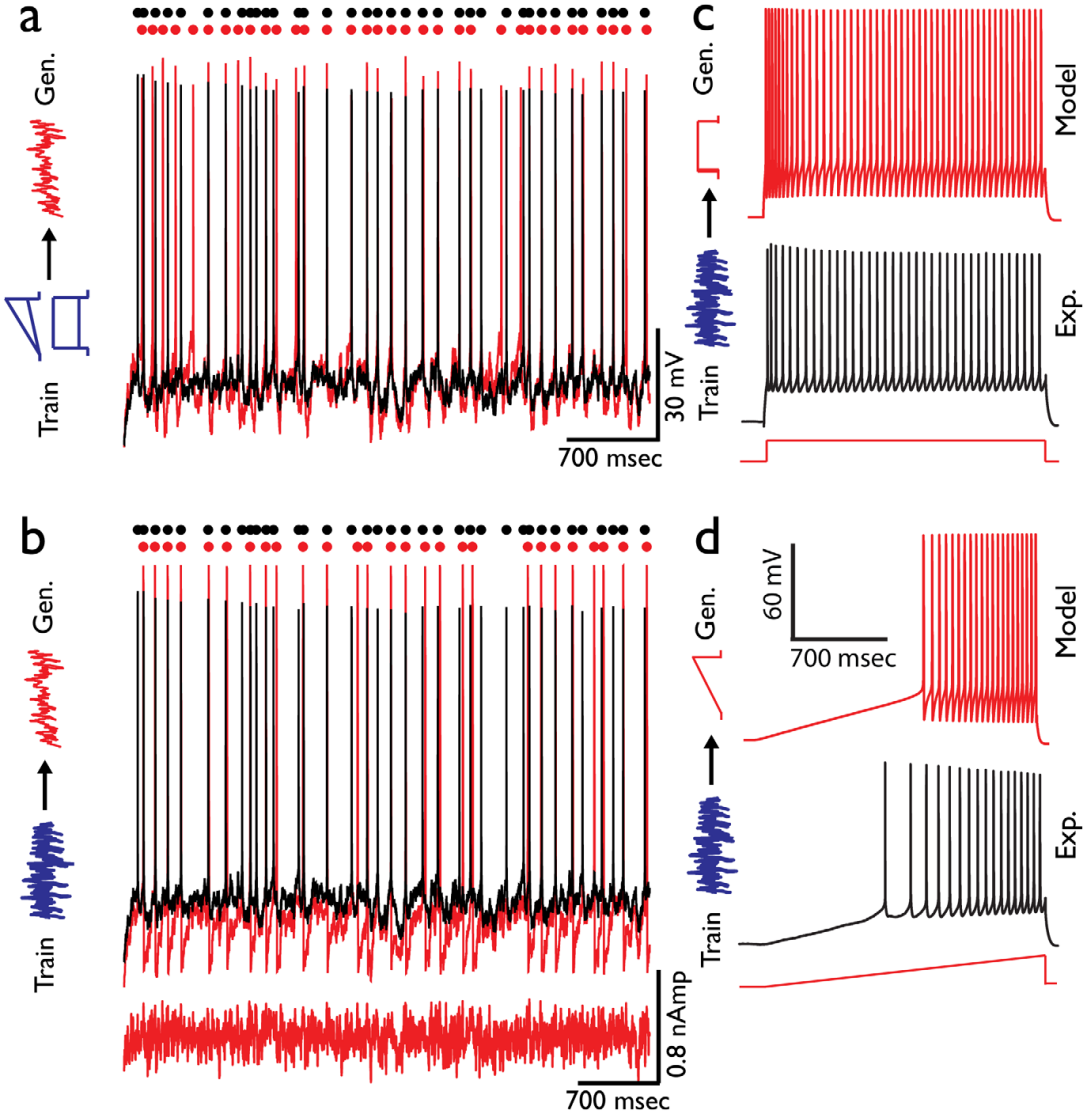
Generalization based on step+ramp stimuli outperforms generalization based on noisy stimuli. (**a**) Models were trained on a combined set of step and ramp stimuli (schematics at left, blue) and tested for generalization on noisy inputs. Experimental response (black) is displayed along with one model response (red). Timing of spikes is highlighted by corresponding color dots at top. GCF value 0.92 (**b**) Generalization of models, trained using the type 1 noisy stimulus, to the type 2 noisy stimulus (black – experimental response; red – model response to type 2 noisy input). GCF value 0.93 (**c**) Generalization of models trained using the type 1 noisy stimulus to step current pulses (black – experimental response, red – response to type 2 noisy input). (**d**) Generalization of models trained on type 1 noisy input to ramp current pulses. Note considerable mismatch in both **c** and **d**.

Step or ramp currents are clearly not likely currents for a neuron to encounter in its natural setting. Thus, we consider in Figure 5 the ability of models constrained with these simple stimuli to predict more physiological noise current injections. We employed the gamma coincidence factor (GCF) [30], [31] in order to measure how well-locked is the timing of model APs generated in response to noisy current injection to the APs recorded experimentally in response to the same current (across multiple experimental repetitions of the current injection). Two different noisy currents were used, one with high mean and low standard deviation (noise type 1) and one with low mean and high standard deviation (noise type 2). We find that models trained on two steps currents and two ramp currents were the best predictors of the experimental AP times that were generated in response to both noisy currents. When comparing the number of APs coincident between the voltage responses derived from two repetitions of the current input, the number of model APs coincident with those of any given experimental repetition was over 90% of the number of APs consistent between two different experimental repetitions (Figure 5a black traces experimental voltage, black dots experimental AP times, red trace model voltage response, red dots model AP times; GCF 0.91±0.03). Very similar accuracy was obtained for the second type of noise current (GCF 0.92±0.04, Figure S2a).

Responses to noise currents can themselves be used to constrain the model by attempting to maximize the temporal fidelity of the model to the experimental AP times. Indeed, models trained on responses to noisy currents achieve a perfect within model accuracy of GCF = 1. Generalization within stimulus type (to the other noisy current type) was also highly successful (Figure 5b GCF 0.95±0.09). However, models trained on noisy currents poorly matched responses to step and ramp current inputs (Figure 5c, 5d average feature error 2.58±0.85 and 2.92±0.97 respectively in experimental SD units). The discrepancies in feature values fell beyond 2.5 experimental SD units, more than twice as much as the between stimulus generalization error of step currents. In addition, the spread of parameter values of successful solutions was very broad (not shown). Thus, the generalization from responses to noise currents to that of simple currents was asymmetric, with the combined step and ramp currents generalizing well to noise currents but not *vice versa*.

We determined the accuracy of generalization from all different training sets to all generalization test sets (Table 1). We find that the combined set of ramp and step stimuli was the most effective in generalizing to responses of both the simple and noise current injections. Among the single stimuli, the step stimulus was the most successful. Additionally, we find that though adding stimulus intensities improves the generalization error, the added benefit of including additional stimulus intensities of the same type in the training set drops after more than three stimulus intensities.

**Table 1:** Summary of generalization errors for different training stimuli.

Training Stimuli	Gen. to Steps Error (mean ± sd)	Gen. to Ramps Error (mean ± sd)	Gen. to Noise type 1 (gamma coinc. factor)	Gen. to Noise type 2 (gamma coinc. factor)
Step (4 intensities, 2 seconds length each)	*0.74±0.18*	1.30±0.82	0.88±0.05	0.87±0.06
Ramp (4 intensities, 2 seconds length each)	2.38±0.69	*0.68±0.15*	0.80±0.05	0.85±0.07
Step+Ramp (2 intensities of step, 2 intensities of ramp, 2 seconds length each)	*0.81±0.35*	*0.92±0.41*	0.91±0.03	0.92±0.04
Noise 1 (8 seconds length)	2.58±0.85	2.92±0.97	*1.00±0.00*	*0.95±0.09*
Noise 2 (8 seconds length)	2.81±0.74	2.63±0.91	*0.94±0.07*	*1.00±0.00*

Left column denotes the five training sets employed: step current pulses only, ramp current pulses only, combined step and ramp current pulses and the two noise inputs. Models were generated by each one of these training sets and tested on four different generalization sets (top row): steps, ramps and type 1 and type 2 colored noise currents (see Materials and methods). Accuracy of generalization to step and ramp currents is measured as the average ± sd of all feature-based errors across all six features, for the acceptable models (lower values indicate greater accuracy). Accuracy of generalization to colored noise current injections is given by the precision of spike timing as quantified by the average gamma coincidence factor value (higher values are more accurate; see Materials and methods). Italicized text indicates within-stimulus generalization, regular indicates between stimulus generalization.

We note that there is no theoretical guarantee that models that generalize well to a certain type of stimulus will also generalize well to different ones. An important class of stimuli are stimuli that continuously sweep through a range of frequencies, sometimes referred to as “chirp” or “zap” stimuli [32]. Though we did not explore the space of such stimuli extensively in our experiments, for the data we have we find that models trained on the combined step and ramp stimuli generalize well to subthreshold frequency sweeps (Figure S3).

**Figure 6:**
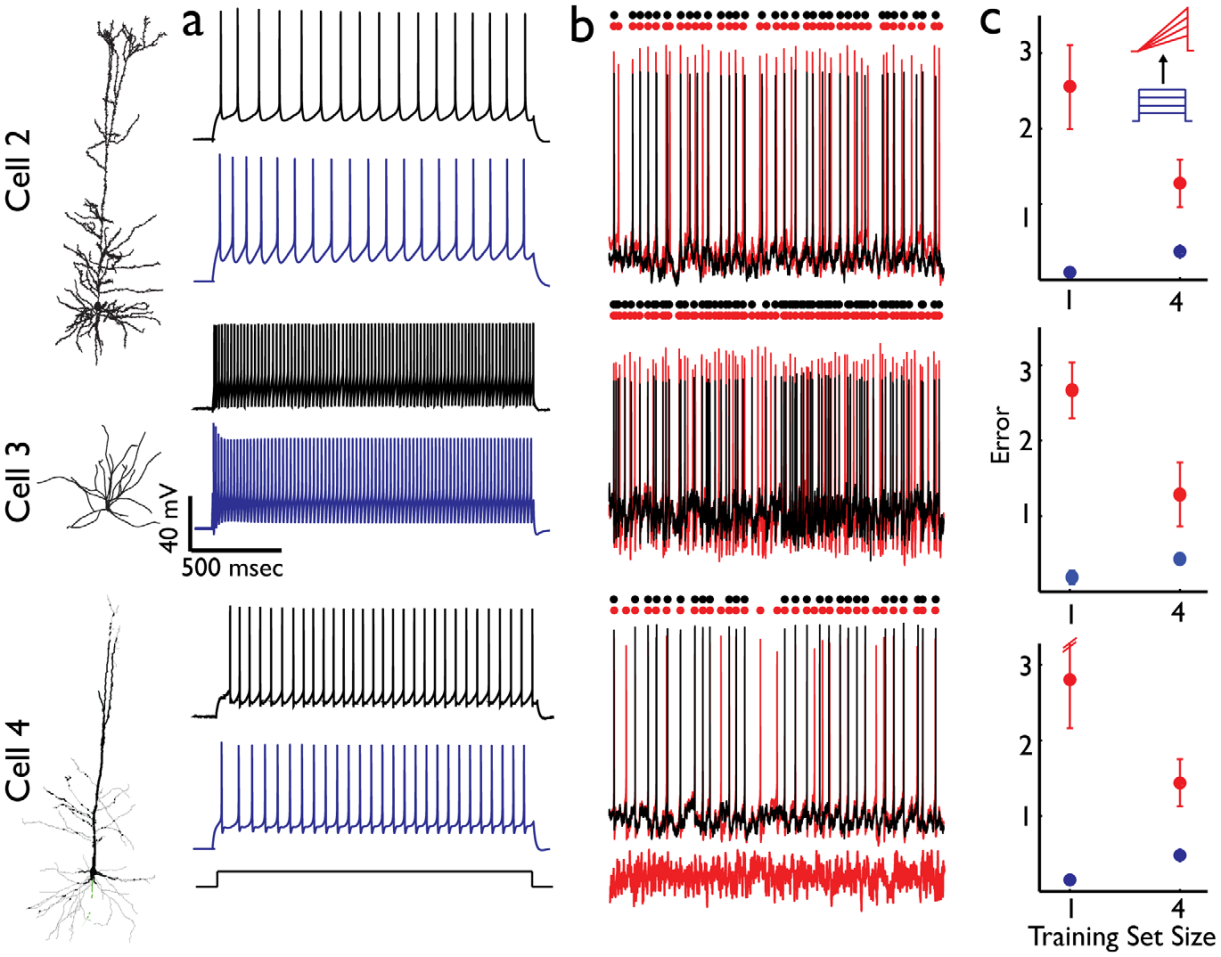
Constraining conductance-based models for different neuron types. (**a**) Experimental response (black traces) to a 2 second long step current pulse (lower trace, black) and model response (blue traces) to the same current pulse; training set consisted of the combined step and ramp currents. (**b**) Generalization was tested on the high mean, low variability type 1 noisy current pulse (bottom grey). Experimental response (black) and one model response (red) are shown along with corresponding color dots indicating timing of APs. GCF values: 0.91, 0.89, 0.92 top to bottom respectively (**c**) Generalization error (red) and training error (blue) for models trained on step currents and generalization tested on ramp currents. Cell 2 - L5 pyramidal cell from a juvenile rat (p16); cell 3 - fast-spiking interneuron, juvenile rat (p16); Cell 4 - pyramidal cell from a young mouse (p34). Corresponding morphology is shown at left.

Results presented so far pertained to models of a rat layer V pyramidal cell. In order to assess the generality of the results we applied the analyses described above to four additional cells. These cells provided examples of different cell types (pyramidal, interneuron), different ages (juvenile, adult) and different animals (rat, mouse). We were able to generate successful CBMs for each of the cells selected (shown in Figure 6a). We found that, in general, the major results highlighted above are consistent across all cells. Namely, the combined set of step and ramp stimuli was the most effective and achieved very high temporal precision values (Figure 6b). The generalization error was reduced as the number of stimuli increased (Figure 6c) and the generalization between stimuli was asymmetrical, with this set capable of matching responses to noisy currents, but not *vice versa*.

## Discussion

To the best of our knowledge, this is the first study to rigorously quantify and successfully incorporate the concept of generalization into the construction of experimentally-constrained conductance-based neuron models (CBMs). Several previous studies have fit models to surrogate data [33], [34], [35], [36] or to experimental data [29], [36], [37] but none have systematically compared the generalization of models derived from different experimental stimuli to novel stimuli. Furthermore, it is the first study showing a systematic successful application of automated parameter constraining of CBMs for a wide set of different stimuli types, different neuron-types and different animals. For the five cells studied, we obtained general results regarding the utility of different stimuli types in constraining CBMs. We believe that the paradigm we propose should hold also for other neuron types (e.g., hippocampal CA1 pyramidal cells) but this requires further exploration.

### Quantitative characterization of the utility of different stimuli

Importantly, by considering the ability of CBMs trained on one stimulus type to predict the responses to a set of different stimuli, we provide a simple and valuable way of measuring the utility of a certain stimulus in generating faithful CBMs. Clearly, evaluating the utility of a given stimulus is of great practical importance not only to those directly involved in biophysical modeling but also to experimentalists as it will provide an objective method of selecting which stimuli to be applied experimentally to a neuron in the limited time of stable recording. Notably, despite its centrality to the modeling effort, this subject has evoked little systematic study, perhaps due to the technical difficulty of generating CBMs that generalize well to experimental data (for surrogate data see ref. [38]). Evaluation of the utility of different stimuli has been performed for simpler biophysical models, such as integrate and fire type models [39]. However, the stimuli found are typically closely tied to the specific phenomenological nature of the model assumed (e.g., a stimulus tailored to accurately measured the AP threshold) and are thus not always applicable to models of a different nature (e.g. models that do not have an explicit parameter for the threshold, such as CBMs).

For the step and ramp currents studied here, we find that multiple suprathreshold intensities of two second long step and ramp currents are required to generate faithful models. For the number of stimulus intensities studied here additional intensities reduce the generalization error (Figures 3,4). Yet, the added benefit of stimuli beyond three intensities diminishes. For the noise currents, we find that ten second long stimuli were sufficient to generate models that generalize well for different noise currents. For each of the stimuli, we use ten repetitions to estimate the intrinsic variability. A combined set of step and ramp stimuli was able to achieve even better generalization (Table 1). Thus, training sets combining different stimuli are expected to be more effective than single stimulus sets in their generalization as we indeed find (see below).

### The success of the step stimulus in generating predictive CBMs

To what do we attribute the success of step stimuli in generalizing to other stimuli? More generally, what could make one stimulus more useful than another in generating models that generalize to a wide variety of stimuli? The intuition behind the success of the step stimulus relies on a combination of the nature of the stimulus itself and single-cell biophysics. The ion-channels expressed by a neuron exhibit a wide range of time constants, from the very brief (less than a millisecond) to the very long (hundreds of milliseconds and more). Different stimuli activate these membrane ion channels to different degrees. If a certain channel is only partially activated by a given stimulus, the contribution of this channel to shaping the model dynamics (and hence the sensitivity of its parameter values) will not be well estimated.

The slow transition through voltage prior to firing an AP elicited by ramp currents strongly inactivates transient currents (e.g., fast inactivating Na^+^ channels). Thus, if only ramp currents are present in the training set, the parameter constraining procedure has no opportunity to “learn” of the possibility of transient activation, leading to an underestimate of the sensitivity of parameter values of transient channels. When this model is challenged with the need to generalize to depolarizing step stimuli, in which the degree of inactivation prior to the first AP is much smaller, the full sensitivity of transient channels comes into play and some of the models fail to generate accurate responses. In contrast, white noise (or noise smoothed by a short correlation time) is essentially a continuous series of transients. This rapid transition between voltage values is ineffective at activating channels with longer time constants. Hence, the sensitivity of channels with long time constants (e.g. slow inactivating potassium channels such as Kp) is underestimated by models trained solely on noise currents. In other words, noise currents are composed only of transient responses and ramp currents lack a strong transient. Step currents, on the other hand, contain both an initial strong transient followed by a sustained level of depolarization. Thus, they are able to activate both transient channels and channels with long time constants, yielding more accurate estimates of their contribution to the overall response of the cell. Note, that had we been dealing with a linear system, white noise would be sufficient to determine its transfer properties and no other stimuli would be required [40]. However, neurons are of course highly nonlinear systems.

The intuitive description above is in line with the quantitative results regarding the effectiveness of generalization from different stimuli i.e., the failure of models trained on ramps to generalize to step currents, (Figure 3), the failure of models trained on noise currents to generalize to steps and ramps (Table 1) and the spread of acceptable parameter values (Figure 3). Notably, the intuitions developed are relevant not only to the specific model itself (as would be the case with phenomenological models) but also to the general understanding of the function of different ion channels in sculpting neuronal dynamics since the models directly incorporate the experimentally derived dynamics of specific channels. In summary, despite the simple and artificial nature of the step current, it is more successful in constraining the dynamics of the neuron than the synaptic-like noisy stimuli that more closely mimic the conditions a neuron might encounter *in-vivo*. Thus, we point out that the similarity to natural conditions should not be the only reason for selecting stimuli. Indeed, one must in addition consider how the stimuli might be used to uncover the underlying biophysical dynamics.

### Quantitative characterization of the parameter space

Mapping the portion of parameter space [29] corresponding to solutions consistent with a given stimulus provides a both intuitive and quantitative view of the effect of different stimuli on model reliability. Different stimuli carve-out different shaped zones in parameter space (see Figure 3). The degree to which two zones overlap is an indication of how well the models will generalize from one to the other, as only those models found in the intersection area are consistent with both. Thus, if one of the stimuli is chosen to train the model, the portion of the area found outside of the intersection area corresponds to models that will fail to generalize to the other stimulus. By combining different stimuli in the training set we obtain different intersections of these zones. Ultimately, we are interested in finding effective intersections that will reduce the space of solutions as efficiently as possible to the intersection of all stimuli measured. Naturally, as we add more and more stimuli at some point the zones will fail to intersect any longer, indicating that we have tasked our models too far and must either choose a different model or less ambitious requirements. Notably, we believe that this provides a very useful framework for tackling the problem of non-uniqueness in the solution space. Importantly, this will allow more detailed exploration of the spread and composition of different membrane channel conductances for a given neuron type and even comparisons between the same neuron type at a different stage of neuronal maturation, or between different neuron types and different species.

In summary, we have demonstrated that, given the experimental response to different stimulus types (and several repetitions of each) and based on the theoretical framework presented here, we can construct faithful CBMs of different neuron types that can accurately predict the responses to both simple and noisy current injections that were not used during model construction. This suggests that the models generated indeed capture the neuron's dynamics. We emphasize that modeling studies should report not only the similarity of models to the data used in their generation (training error) but should also reserve some of their data for examining the generalization (or predictive) quality of the models. We note however that there is no guarantee that models that generalize well to a certain stimulus will also generalize well to other stimuli and this issue requires more careful exploration with many stimuli. Our development of a framework to quantitatively test the utility of different stimuli and our finding that some stimuli are more advantageous in constraining CBMs than other stimuli has prompted us to start exploring experimentally and theoretically the effectiveness of more sophisticated stimulus protocols in constraining neuronal models. Ultimately, the goal is to find the optimal (and minimal) set of stimuli that ensure accurate generalization to a wide set of diverse stimuli. There are numerous possible options for the different forms of stimuli that could be injected within a fixed time, for instance frequency sweeps that explore frequency response and resonant properties of neurons [32], [41] or more complicated noisy stimuli that alternate between different noise parameters [42], [43]. This is a subject that is presently under active pursuit.

## Materials and Methods

### Ethics statement

Wistar rats (17–19 days old) and one x98 mouse [44] were quickly decapitated according to the Swiss national and institutional guidelines.

### Slice preparation and cell identification

The brain was carefully removed and placed in ice-cold artificial cerebrospinal fluid (ACSF). 300 mm thick parasaggital slices were cut on a HR2 vibratome (Sigmann Elektronik, Heidelberg, Germany). Slices were incubated at 37°C for 45–60 min and then left at room temperature until recording. Cells were visualized by infrared differential interference contrast videomicroscopy utilizing a VX55 camera (Till Photonics, Gräfeling, Germany) mounted on an upright BX51WI microscope (Olympus, Tokyo, Japan). Cells were patched in slices ∼1.8 mm lateral to the midline and above the anterior extremity of the hippocampus ±0.8 mm, corresponding to the primary somatosensory cortex [45], [46], [47]. Thick tufted layer 5 PCs (rat and mouse) were selected according to their large soma size and their apparent large trunk of the apical dendrite. Layer 6 fast-spiking interneurons were selected according to their multipolar soma shape. Care was taken to use only “parallel” slices, i.e. slices that had a cutting plane parallel to the course of the apical dendrites and the primary axonal trunk. The cell type was confirmed by biocytin staining revealed by standard histochemical procedures [48].

### Chemicals and solutions

Slices were continuously superfused with ACSF containing (in mM) 125 NaCl, 25 NaHCO3, 2.5 KCl, 1.25 NaH_2_PO_4_, 2 CaCl_2_, 1 MgCl_2_, and 25 D-glucose, bubbled with 95% O2 – 5% CO2. The intracellular pipette solution (ICS) contained (in mM) 110 K-gluconate, 10 KCl, 4 ATP-Mg, 10 phosphocreatine, 0.3 GTP, 10 N-2-hydroxyethylpiperazine-N9-2-ethanesulfonic acid (HEPES), and 13 biocytin, adjusted to a pH 7.3–7.4 with 5 M KOH. Osmolarity was adjusted to 290–300 mosm with D-mannitol (35 mM). The membrane potential values given were not corrected for the liquid junction potential, which was approximately −14 mV. All chemicals were from Sigma-Aldrich (Steinheim, Germany) or Merck (Darmstadt, Germany).

### Electrophysiological recordings

Whole cell recordings (1–3 cells simultaneously) were performed with Axopatch 200B amplifiers (Molecular Devices, Union City, CA) in the current clamp mode at a bath temperature of 34±1°C during recording. Data acquisition was performed with an ITC-1600 board (Instrutech Co, Port Washington, NY), connected to a Macintosh running a custom written routine under IgorPro (Wavemetrics, Portland, OR). Sampling rates were 10 kHz, and the voltage signal was filtered with a 2 kHz Bessel filter. Patch pipettes were pulled with a Flamming/Brown micropipette puller P-97 (Sutter Instruments Co, Novato, CA) and had an initial resistance of 3–4 MW. During recording the series resistance was 10, 10, 11, 17, or 22 MW and bridge balanced. Miniature excitatory postsynaptic potentials (mEPSPs) were blocked with 10 mM CNQX and occasionally with 40 mM AP5.

### Stimulation protocols

Three different types of stimuli were applied. Stimuli were scaled with a constant factor *k* ∈ (1, 2, 2, 2.5, 3) so that the cells fired with moderate mean frequencies of 2–20 Hz, high enough to obtain enough spikes for analysis, yet low enough not to over stimulate the cells and shorten their life span. Six depolarizing step currents of 2 s duration and increasing amplitudes (100–225×*k* pA) were applied. Five depolarizing ramp currents, 2 s rising phase (from 0 to 125–250×*k* pA) and symmetrically decaying falling phase, were injected (only rising phase was used in this study). In addition, we apply Ornstein-Uhlenbeck [49] (OU) colored noise processes that are considered to represent the current that might arrive at the soma of a cell as a result of the summation of the activation of many synapses in the cell's dendritic arbor [50]. We employ two different 20 s long OU processes with identical correlation time (2 ms) but different statistics. One is generated with a mean of 50×*k* pA and SD of 100×*k* pA (hereby referred to as noise type 1). The other mirrors this process by having a mean of 100×*k* pA and SD of 50×*k* pA (hereby referred to as noise type 2). We repeatedly inject the different currents in order to measure response variability. Each stimulus was repeated 10–20 times.

### Neuron model

All simulations were performed in the NEURON simulation environment [51]. The morphology of 5 cortical neurons from rat and mouse somatosensory cortex was derived from reconstruction of *in-vitro* stained cells. The number of compartments employed differed from cell to cell, all cells contained more than a hundred compartments. Specific axial resistance was 150 Ωcm and capacitance was 1 µF. The following ion channels were assumed to be present in the membrane of the modeled soma: Transient sodium channel-Na, Delayed potassium channel-Kd, Slow inactivating persistent potassium channel-Kp, fast non-inactivating potassium channel Kv3.1 channel, high-voltage-activated calcium channel Ca, calcium dependent K channel - SK, Hyperpolarization-activated cation current – Ih, M-type potassium channel Im, for full details see ref. [27]. The dynamics of these channels were described using Hodgkin and Huxley formalism [1]. As all the experimental recordings in this work were performed from the cell's somata and for the sake of simplicity, the modeled dendrites were assumed to be passive. The maximal conductance of all eight channels along with the leak reversal potential and leak conductance in the soma and dendrite served as free parameters, yielding a total of eleven free parameters in the model. The allowed range for the conductances can be found in ref. [27].

### From data to conductance-based model

An overview of the procedure by which we generate conductance-based models (CBMs) from an experimental data set is presented in Figure S1. We begin by recording the responses (Figure S1a) of the cell to intracellular current injection. Responses are then analyzed by the extraction of a set of features (Figure S1b), which are used to generate feature-based distance functions (see below). Next, we use the reconstructed morphology (Figure S1c) to generate the compartmental model of that cell and assume a set of 8 ion channels to be present in the soma membrane of the model cell. Together the reconstructed morphology and the assumed ion channels compose the model skeleton. When combined with a set of specific values for the free parameters they together constitute a single CBM for that neuron.

A stochastic optimization procedure is employed to constrain the parameters of the model in accordance with the experimental data. We employed a multiple objective optimization (MOO) algorithm which operates by genetic algorithm optimization [52]. The algorithm evaluates 300 sets of parameter values in parallel and iteratively seeks to reduce the error, which measures the discrepancy between model and experiment (Figure S1d). As the algorithm is stochastic in nature, we repeat the optimization procedure ten times in order to reduce the chance that the optimization procedure fails to converge. Thus, at the end of the optimization procedure, 3000 parameter sets, i.e. 3000 tentative models of the cell are obtained along with their corresponding error values. We then choose only those models that pass the acceptance criterion of a model-experiment mismatch no greater than two SD in each feature (Figure S1e). Ultimately, we end up with a set of models that closely match the experimental voltage responses (Figure S1f).

### Distance functions

The discrepancy between the target experimental data (a train of spikes in response to a set of current stimuli) and model simulation of the response was measured using feature-based distance functions [27]. Features to be fitted were extracted from the firing response of the neuron (e.g. number of action potentials (APs), spike height). The value of each feature was derived from the experimental responses. The model response to the same stimulus was then analyzed in an identical fashion. The model-to-experiment distance value, for this feature, was measured by the distance of the model feature value from the experimental mean, in units of experimental SD. These distance functions have two main advantages. First, they address experimental variability by considering the distance of a model in relation to the experimental SD. Second, they are expressed in well defined, *not arbitrary*, units.

For step pulses, we employ a set of six features: the number of action potentials (APs) during the pulse, the time to the first AP from stimulus onset, the accommodation index (a measure of the accommodation in the rate of APs during the stimulus [27]), the width of an AP at half height, the average height of an AP, and the average depth of the after hyperpolarization (AHP) as defined by the minimal voltage point. For ramp currents, as the height of APs decreases during the stimulus response, we considered not only the average height of APs and the depth of AHPs but also the slope of a linear fit to the change as an additional feature. For the noise stimuli we do not use feature-based distance functions, but rather the gamma coincidence factor [30], [31] - an index measuring the coincidence of AP times in relation to the neuron's intrinsic reliability. The index is normalized from 0 to 1, a value of 0 indicates that a model does no better than a Poisson train and a value of 1 indicates that the model and experimental repetitions have as many coincident spikes on average as do two experimental repetitions. Note, that in this context the objective of optimization is to maximize this value.

### Assessing utility of different stimuli

In order to assess the utility of different stimuli in generating neuron models that generalize well both within stimulus and across stimuli we generate models with training sets that are equally matched in terms of the length of the experimental data. Namely, we consider four different training sets: step current pulses only (four intensities of two second long step currents), ramp current pulses only (four intensities of two second long ramp currents), combined step and ramp currents (two intensities of two second long step currents and two intensities of two second long ramp currents) and noise currents (eight seconds of OU noise process current injection). For each of these training sets, we test the generalization of the model to four different conditions: step currents, ramp currents and two different noise currents. Five intensities of step and ramp currents can be potentially employed to both train and test generalization. Stimuli used during the parameter constraining process (e.g. the four step currents used by the first training set) are excluded from the generalization test.

### Multiple objective optimization

As we typically employ several feature-based distance functions per stimulus and we often use more than one stimulus for the optimization, we obtain multiple distance function values for each model-experiment comparison. To obtain a single value a weight vector is used to sum all the different distance functions. Here we employ a different approach termed multiple objective optimization (MOO) [53]. This approach maintains the multiple distance measures and does not employ a weight vector. Instead, the relation between distance measures is that of *domination*: solution *i* is said to dominate solution *j* if for all distance functions the values of solution *i* are no greater than those of solution *j* and for at least one distance function the value of solution *i* is strictly lower than that of solution *j*. The purpose of a multiple objective optimization procedure is to find the best possible tradeoffs between the distance functions, termed the *Pareto front*.

### Optimization algorithm

We employ a genetic algorithm (GA) based optimization algorithm designed for multiple objective optimization named NSGA-II [52]. This algorithm is an elitist (GA) with a parameter-less diversity preserving mechanism. We custom implemented this algorithm in NEURON. We find that the algorithm almost always converges after 1000 iterations of evaluation of the full set of parameter values. As a safety factor, 1500 iterations were used. We repeated each given optimization ten times.

### Analysis of solution space

The spread of successful solutions in parameter space for a given stimulus type can be explored by simply marking the location of each point corresponding to a solution. However, it is difficult to determine in this fashion whether a certain region of parameter space is consistent with more than one stimulus as the points themselves will almost surely not coincide. An additional disadvantage is that many of the solutions are the result of the same optimization run and thus contain artificial correlations due to the closely linked nature of solutions generated by a single optimization run. To overcome these two difficulties we complement our analysis by additional simulation of the response of a large set of points placed on a high-dimensional grid [29] to all (step and ramp) stimuli used in the experiments. This approach is extremely computationally expensive. However, it overcomes the above-mentioned difficulties: as the same points are simulated for all conditions, one can easily ascertain which are the conditions consistent with each point. Secondly, as all points are generated on the grid there are no unknown artificial correlations between them. Lastly, this approach allows visualization of projections of the space of solutions consistent with each stimulus (see Figure 2).

## Supporting Information

Figure S1 **Successful solution conductance values for all ion channels.** (**a**) Normalized conductance values for all eleven ion channels modeled. Black dots - models trained on one current step; red dots - model trained on four step current stimuli. Note that for most conductances the range of acceptable values decreases with the number of stimuli. (**b**) Corresponding plot for models constrained using either one (black) or four (red) ramp stimuli.
(TIF)

Figure S2 **Generalization to second noise type.** (**a**) Models were trained on a combined set of step and ramp stimuli (schematics at left, blue) and tested for generalization on a high mean low standard deviation noisy current injection (type 2, bottom). Red trace shows model response to stimulus, black trace one experimental trace. AP times highlighted by correspondingly colored dots. (**b**) Corresponding plot for models trained on the low mean high standard deviation noisy current injection (type 1). Blue trace shows model response, black experimental; colored dots highlight AP times.
(TIF)

Figure S3 **Generalization of model constrained by step and ramp stimuli to “chirp” stimuli.** a. Experimental subthreshold response (black line) of layer 5 pyramidal cell shown in Figure 1–5 to sinusoidal stimuli of increasing frequency with time (“chirp” stimuli). The generalization result of a model of that cell, trained on the combined step and ramp stimulus set is depicted in red. b. Responses to five experimental repetitions of the chirp stimulus. For each of the repetitions, the height of successive local peaks was normalized to the height of the first peak. The attenuation of the peaks with time corresponds to increasing chirp frequency. The mean of the experimental traces is shown in thick black, the five individual repetitions experimental plots in thin gray and model response is shown in thick red. Note the accurate, but not perfect match between model and experiments. c. Due to the lack of experimental suprathreshold chirp responses, we generated surrogate data for these stimuli by first fitting a model of the same pyramidal cell, using step and ramp current injections, then generating surrogate data from that neuron by simulating injections of different stimuli including suprathreshold chirp stimuli and collecting surrogate data from the model neuron. Later, new acceptable models were generated from the surrogate step and ramp stimuli data, and their generalization to the surrogate suprathreshold chirp stimuli data was tested. Voltage traces for the surrogate chirp stimuli are shown in black and AP times marked above as circles (note that APs were cut). Superimposed in red is the response of model the for the chirp stimulus, that was generated from the surrogate data. In the bottom, marked by Amp. 2, AP times are shown with the same convention for a stronger amplitude chirp. Many of the AP times were accurately reproduced.
(TIF)
