# Correction: Effective Stimuli for Constructing Reliable Neuron Models

**DOI:** 10.1371/annotation/c002fbe1-712c-4608-9747-f1185f0b7cf4

**Published:** 2013-06-06

**Authors:** Shaul Druckmann, Thomas K. Berger, Felix Schürmann, Sean Hill, Henry Markram, Idan Segev

In the Materials and Methods subsection "From data to conductance-based model" and in the legend of Figure 2, all references to Figure S1 should instead refer to Figure 1.

