# Supplementary figures and images for: Effective Stimuli for Constructing Reliable Neuron Models
Source: PLoS Comput Biol. 2011 Aug 18;7(8):e1002133. doi: 10.1371/journal.pcbi.1002133 (PMC3158041; doi:10.1371/journal.pcbi.1002133)

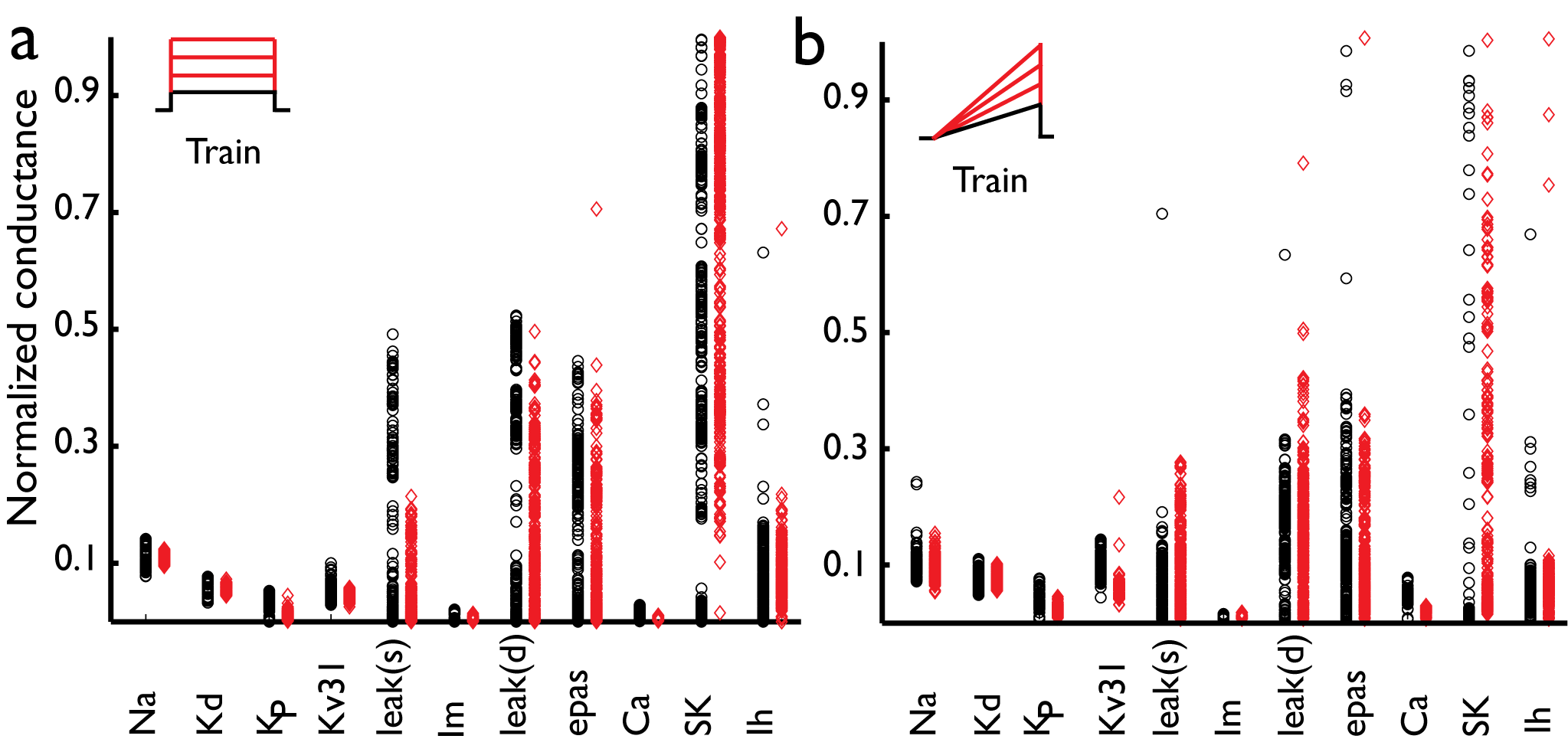

Supplement: Figure S1 — Successful solution conductance values for all ion channels. (a) Normalized conductance values for all eleven ion channels modeled. Black dots - models trained on one current step; red dots - model trained on four step current stimuli. Note that for most conductances the range of acceptable values decreases with the number of stimuli. (b) Corresponding plot for models constrained using either one (black) or four (red) ramp stimuli. (TIF) [file pcbi.1002133.s001.tif]

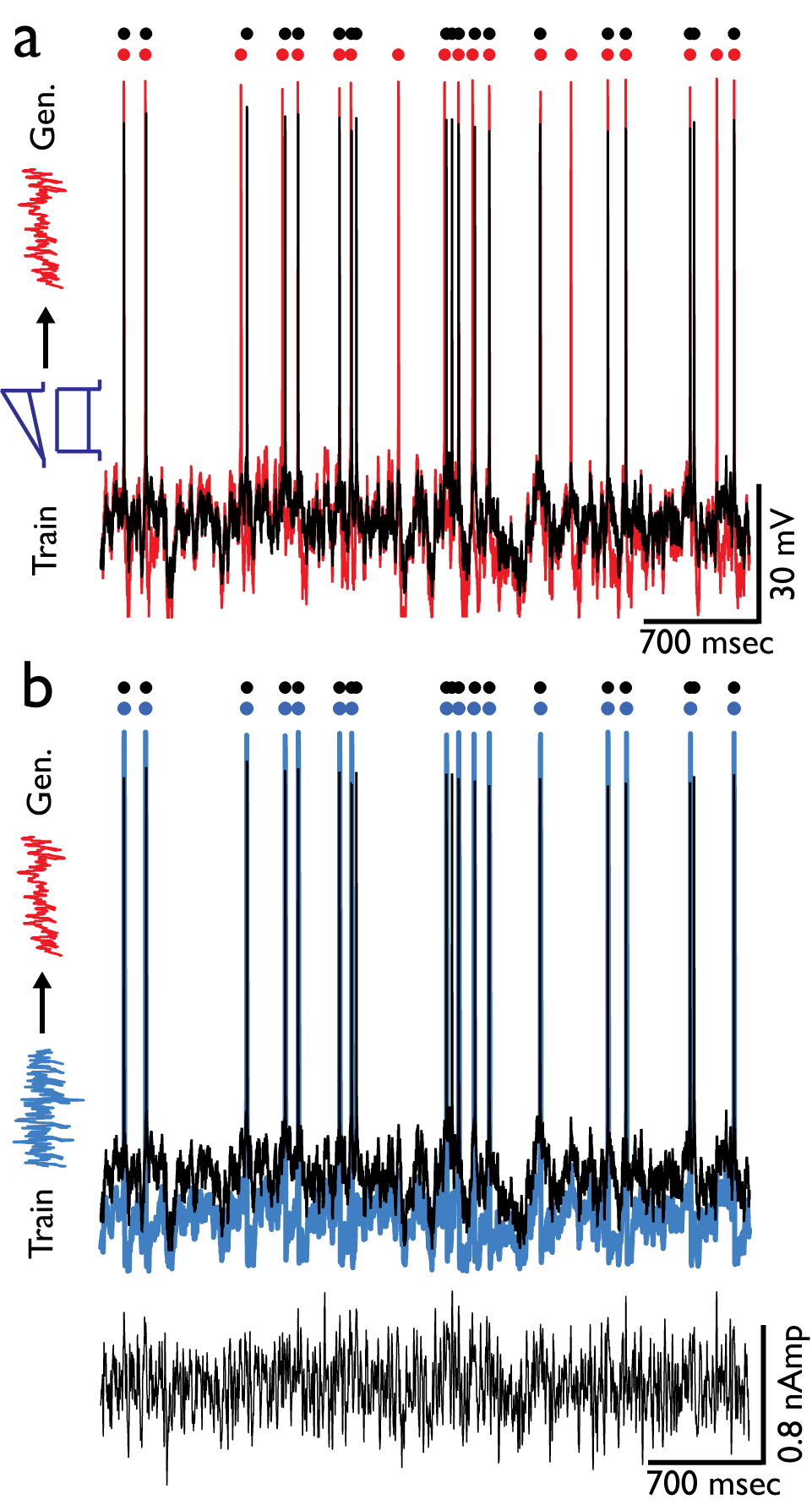

Supplement: Figure S2 — Generalization to second noise type. (a) Models were trained on a combined set of step and ramp stimuli (schematics at left, blue) and tested for generalization on a high mean low standard deviation noisy current injection (type 2, bottom). Red trace shows model response to stimulus, black trace one experimental trace. AP times highlighted by correspondingly colored dots. (b) Corresponding plot for models trained on the low mean high standard deviation noisy current injection (type 1). Blue trace shows model response, black experimental; colored dots highlight AP times. (TIF) [file pcbi.1002133.s002.tif]

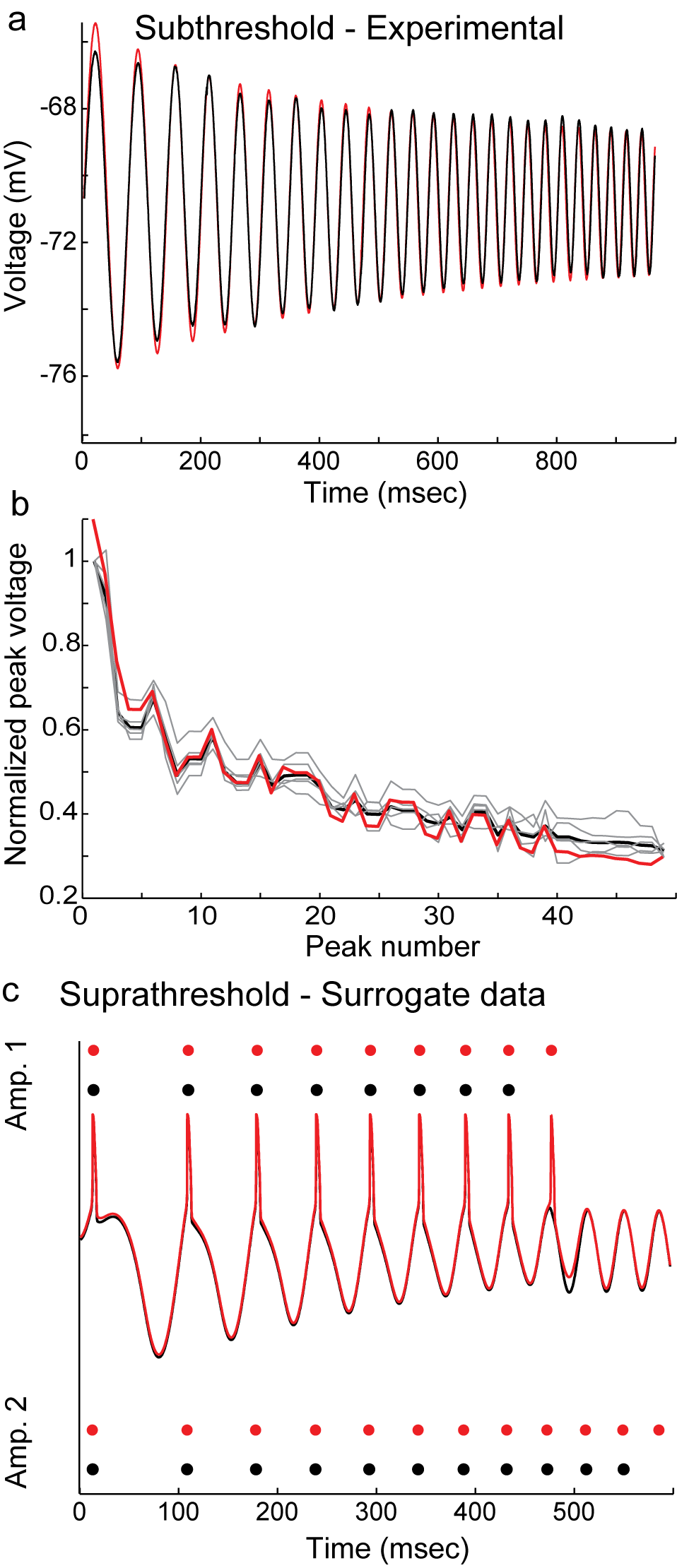

Supplement: Figure S3 — Generalization of model constrained by step and ramp stimuli to “chirp” stimuli. a. Experimental subthreshold response (black line) of layer 5 pyramidal cell shown in Figure 1– 5 to sinusoidal stimuli of increasing frequency with time (“chirp” stimuli). The generalization result of a model of that cell, trained on the combined step and ramp stimulus set is depicted in red. b. Responses to five experimental repetitions of the chirp stimulus. For each of the repetitions, the height of successive local peaks was normalized to the height of the first peak. The attenuation of the peaks with time corresponds to increasing chirp frequency. The mean of the experimental traces is shown in thick black, the five individual repetitions experimental plots in thin gray and model response is shown in thick red. Note the accurate, but not perfect match between model and experiments. c. Due to the lack of experimental suprathreshold chirp responses, we generated surrogate data for these stimuli by first fitting a model of the same pyramidal cell, using step and ramp current injections, then generating surrogate data from that neuron by simulating injections of different stimuli including suprathreshold chirp stimuli and collecting surrogate data from the model neuron. Later, new acceptable models were generated from the surrogate step and ramp stimuli data, and their generalization to the surrogate suprathreshold chirp stimuli data was tested. Voltage traces for the surrogate chirp stimuli are shown in black and AP times marked above as circles (note that APs were cut). Superimposed in red is the response of model the for the chirp stimulus, that was generated from the surrogate data. In the bottom, marked by Amp. 2, AP times are shown with the same convention for a stronger amplitude chirp. Many of the AP times were accurately reproduced. (TIF) [file pcbi.1002133.s003.tif]
